# An isogenic cell line panel for sequence-based screening of targeted anticancer drugs

**DOI:** 10.1016/j.isci.2022.104437

**Published:** 2022-05-23

**Authors:** Ashley L. Cook, Nicolas Wyhs, Surojit Sur, Blair Ptak, Maria Popoli, Laura Dobbyn, Tasos Papadopoulos, Chetan Bettegowda, Nickolas Papadopoulos, Bert Vogelstein, Shibin Zhou, Kenneth W. Kinzler

**Affiliations:** 1 Ludwig Center for Cancer Genetics and Therapeutics, Johns Hopkins University School of Medicine, Baltimore, MD 21205, USA; 2 Cellular and Molecular Medicine Program, Johns Hopkins University School of Medicine, Baltimore, MD 21205, USA; 3 Department of Oncology, Johns Hopkins Medical Institutions, Baltimore, MD 21287, USA; 4 Sidney Kimmel Cancer Center, Johns Hopkins University School of Medicine, Baltimore, MD 21205, USA; 5 Department of Neurosurgery, Johns Hopkins University School of Medicine, Baltimore, MD 21205, USA; 6 Howard Hughes Medical Institute, Johns Hopkins University School of Medicine, Baltimore, MD 21205, USA; 7 Sol Goldman Pancreatic Cancer Research Center, Johns Hopkins University School of Medicine, Baltimore, MD 21205, USA

**Keywords:** Biochemistry, Cancer, Biochemical analysis

## Abstract

We describe the creation of an isogenic cell line panel representing common cancer pathways, with features optimized for high-throughput screening. More than 1,800 cell lines from three normal human cell lines were generated using CRISPR technologies. Surprisingly, most of these lines did not result in complete gene inactivation despite integration of sgRNA at the desired genomic site. A subset of the lines harbored biallelic disruptions of the targeted tumor suppressor gene, yielding a final panel of 100 well-characterized lines covering 19 frequently lost cancer pathways. This panel included genetic markers optimized for sequence-based ratiometric assays for drug-based screening assays. To illustrate the potential utility of this panel, we developed a high-throughput screen that identified Wee1 inhibitor MK-1775 as a selective growth inhibitor of cells with inactivation of *TP53*. These cell lines and screening approach should prove useful for researchers studying a variety of cellular and biochemical phenomena.

## Highlights

• Creation of an isogenic cell line panel representing the loss of 19 cancer pathways
• HTS confirmed MK-1775 as a selective inhibitor of cells with loss of *TP53*
• These cell lines are useful for studying a variety of cellular biochemical phenomena

## Graphical abstract

**Figure:**
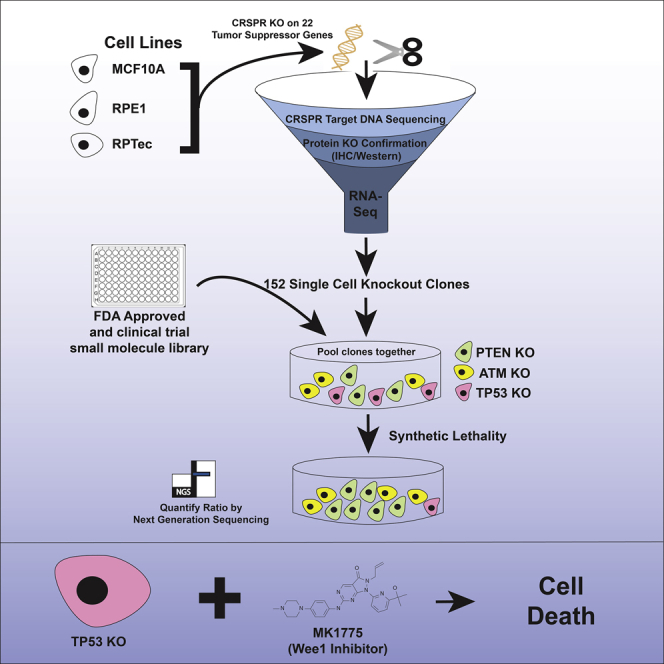

## Introduction

Recent advances in chemical synthesis techniques and robotics have led to an expansion in the availability of small molecule libraries (Clark et al., 2009; Gerry and Schreiber, 2018). With the availability of curated libraries containing more than a million compounds, screening emphasis has shifted to identifying good targets and robust screens to efficiently exploit these libraries (Hatzis et al., 2014; Mullard, 2019). High-throughput screening (HTS) assays can broadly be divided into biochemical and cell-based assays. Biochemical assays enjoy the advantages of low cost, facile scaling, specificity of measured outcome, and the ability to incorporate rigorous controls (Inglese et al., 2007). However, not all pathways, cellular functions, or phenotypes can be adequately captured in biochemical assays. For example, cell-based assays have the advantage of directly identifying compounds that produce the desired biological effect via known or unknown mechanisms (Pech et al., 2019).

The unprecedented progress in defining the cancer genome gave rise to hope for the development of new targeted cancer therapeutics. This hope was largely driven by early success of targeted therapies that inhibited the function of oncogenic driver mutations (Druker et al., 2001). However, while the typical adult solid tumor harbors 3 or more driver gene mutations, most of these mutations affect tumor suppressor genes, with many tumors lacking even a single oncogene mutation (Vogelstein et al., 2013). Even when effective therapies for targeting oncogenes are found, resistance to monotherapy is almost guaranteed in patients with major tumor burden (Diaz et al., 2012; Engelman and Settleman, 2008; Garraway and Lander, 2013). The optimal strategy to overcome this resistance is to treat patients with combinations of drugs targeting different cancer growth mechanisms (Diaz et al., 2012). But as noted above, more than one oncogene mutation is unusual in most common cancer types.

Effective strategies for targeting loss of functions associated with tumor suppressor gene (TSG) mutations would substantially increase the number of therapeutically addressable pathways. Unfortunately, to date, only one FDA-approved therapy specifically exploits a TSG loss of function mutation (Lord and Ashworth, 2017; Zhao and DePinho, 2017). This therapy, as well as other approaches for targeting the loss of function associated with TSG mutations is based on the concept of synthetic lethality or essentiality (Hartwell et al., 1997; Kaelin, 2005). This concept was originally described in yeast, and a key aspect of assigning specificity to synthetic lethality is the availability of isogenic cells differing only in a single genetic alteration (Torrance et al., 2001; Wang et al., 2017). CRISPR-based technologies allow the creation of such lines in human cells.

In response to these issues, we created a human isogenic cell line panel targeting 19 critical genes inactivated in cancer. Each of these lines was engineered using CRISPR-based methods to disrupt a single tumor suppressor gene, and each contained a unique genetic barcode to permit multiplex screening. For each cell line, multiple orthogonal assays were used to validate successful gene disruption. Moreover, the panel was constructed from three distinct normal cell lines to ensure the generality of observed affects. And, finally, a sequence-based ratiometric assay was designed from this panel that incorporates numerous internal controls to maximize the reliability and sensitivity of the screening process.

## Results

### CRISPR-Cas9 creation of the isogenic cell line panel targeting critical tumor suppressor gene pathways

We first sought to create a resource for screening compounds active in critical cancer pathways. We focused on 22 pathways that were collectively altered in greater than two-thirds of the cancers as assessed in multiple large scale sequencing efforts (Tables 1 and S1). In total, 22 TSGs and 3 control genes with known small molecule sensitivities (Table S2) were chosen for targeting by CRISPR-mediated knockouts (Table 1). Typically, 6 gRNAs were employed for each target gene (range 6–12) with three chosen from published studies and three designed *de novo*, targeting either known mutation sites identified from the COSMIC database or early exons within the gene (Sanjana et al., 2014; Smurnyy et al., 2014) (Tables 1 and S3). A total of 162 gRNAs were individually introduced into lentivirus constructs for gene targeting. Three distinct non-cancerous epithelial cell lines, RPE1 (retinal), MCF10A (breast), and RPTec (renal), were targeted. All three lines have a predominately normal karyotype and the only known genetic alteration among the three lines was a homozygous deletion of the *CDKN2A* gene in MCF10A (Jonsson et al., 2007). After transduction with lentiviral CRISPR-Cas9 and puromycin selection, over 1,800 individual CRISPR-targeted single cells were picked and expanded for subsequent characterization.

**Table 1:** Cancer pathway knockout panel

Gene	Chromosome	# gRNA designed	RPE1	MCF10A	RPTec	Core pathway
APC	5	12	2	3	1	APC Signaling Pathway
ARID1A	1	6	2	2	0	Chromatin Modification
ATM	11	6	2	0	2	DNA Damage Control
ATRX	X	12	0	3	1	Chromatin Modification
BRCA2	13	6	0	0	0	DNA Damage Control
CDKN2A	9	6	1	0	3	Cell Cycle/Apoptosis
CDKN2C	1	6	2	2	0	Cell Cycle/Apoptosis
DAXX	6	6	0	3	0	Chromatin Modification; Cell Cycle/Apoptosis
EZH2	7	6	3	3	2	Chromatin Modification
FBXW7	4	6	0	0	0	NOTCH Signaling Pathway
KMT2D/MLL2	12	6	1	1	0	Chromatin Modification
MLH1	3	6	0	2	1	DNA Damage Control
MSH2	2	6	3	2	2	DNA Damage Control
MSH6	2	6	3	1	3	DNA Damage Control
NF1	17	6	1	2	0	RAS Signaling Pathway
NOTCH1	9	6	3	2	3	NOTCH Signaling Pathway
PTCH1	9	6	2	2	0	Hedgehog Signaling Pathway
PTEN	10	6	2	1	3	PI3K Pathway Signaling
SMARCB1	22	6	0	0	0	Chromatin Modification
STAG2	X	6	3	3	3	DNA Damage Control
TET2	4	6	3	3	0	Chromatin Modification
TP53	17	6	3	2	3	Cell Cycle/Apoptosis; DNA Damage Control
MTAP^a^	9	6	0	0	0	Control (Table S2) and Passenger Target
TK1^a^	9	6	0	0	0	Control (Table S2)
HPRT^a^	X	6	0	0	0	Control (Table S2)
Totals			**36**	**37**	**27**	

100 cell lines composing the Cancer Pathway Knockout Panel detailed by targeted genes and cell line background.

Details of gRNA can be found in Table S3. Cellular Processes with Core Pathways in parentheses were defined as in Vogelstein et al. (2013).

a Control KOs are not part of the cancer core panel but are available as described in the Materials and Methods.

### Genetic characterization of candidate knockout lines

**Figure 1:**
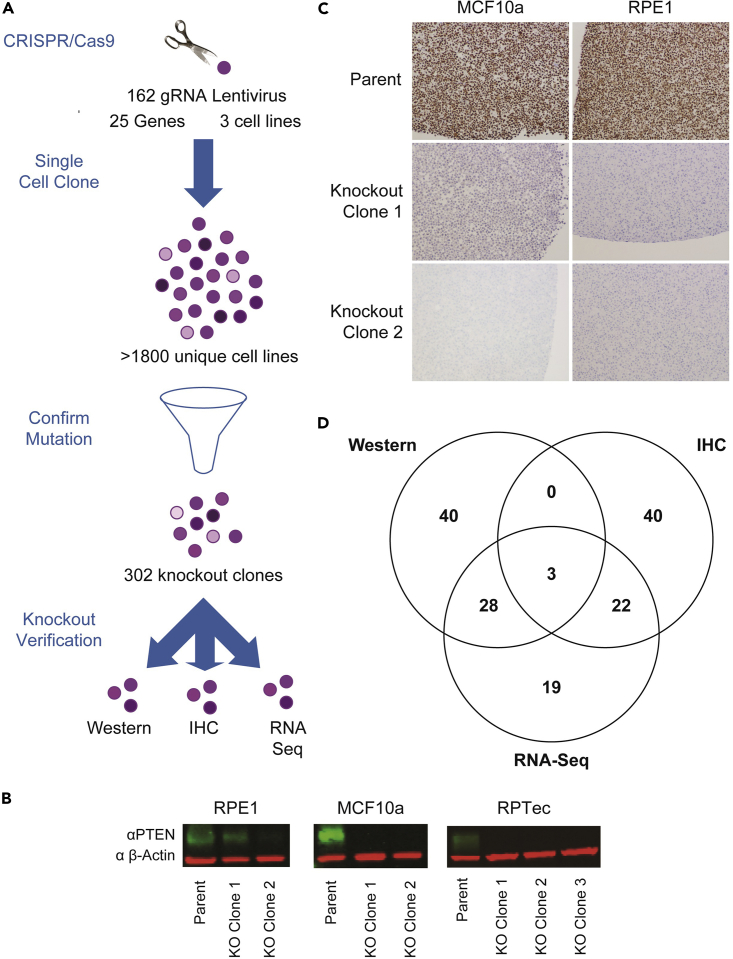
Design and validation of knockout isogenic cell line panel (A) Knockout cell lines were generated by targeting each gene individually with 6 or more gRNAs for a total of 162 gRNAs covering 25 genes in 3 cell lines (RPE1, MCF10a, and RPTec) (Tables 1 and S3). Over 1,800 single cells were selected and expanded, then targeted NGS employed to verify bialleic out of frame insertion or deletion mutations, of which, 302 of these new-found cell lines met the criteria. (B) Representative western blot expression of *PTEN* (green) and *β-actin* (red) in RPE1, MCF10a, and RPTec cell lines. The first knockout cell line in RPE1 is not a true knockout, with *PTEN* protein present, while the other knockout cell lines show no *PTEN* protein expression. Only the regions of the gel relevant to PTEN and beta actin staining and the revelant clones are shown. (C) Representative IHC staining of *ARID1A* protein in RPE1 and MCF10a cell lines demonstrating protein loss. (D) 302 Cell lines with confirmed genetic mutation underwent secondary knockout verification of a combinatory of protein and/or RNA. A total of 152 cell lines passed protein and/or knockout validation with the number of cell lines passing each method indicated. Circles in Venn diagram are not drawn to scale to improve readability.

A massively parallel sequencing approach was used to assess targeting and ensure that essentially all of the cells within any chosen cell line had the expected genotype. For this purpose, a SafeSeqS approach was implemented which utilizes unique molecular barcodes to reduce errors from PCR or sequencing (Kinde et al., 2011). For each gRNA, two distinct sets of primer pairs were designed to cover the targeted region. In total, 324 PCR primer pairs were designed and used to amplify the 162 gRNA genomic target regions (Table S4). This analysis confirmed successful gene disruption in only 302 of the greater than 1,800 lines tested. Though one might have expected a higher fraction of successfully targeted lines based on the previous successes of functional screens (Ling et al., 2020), our criteria for gene disruption were particularly stringent: both alleles had to contain out-of-frame insertions or deletions that could not be readily “rescued” by skipping an exon during splicing. Moreover, we used high-depth sequencing and required that the fraction of reads containing an intact targeting site was <1%. In the 302 lines chosen on the basis of the sequencing results, the deletions ranged from 1 bp to 38 bp and the insertions ranged from 1 bp to 43 bp (Figure S1). Over 31% of cell lines harbored a single base pair insertion or deletion, and an additional 9% of the lines harbored 2 bp insertion or deletion (Figure S1). The targeting success rate varied across genes and cellular backgrounds. Overall, we successfully identify cell lines with biallelelic gene inactivation in 22 of the 25 targeted genes in one or more cellular background, covering 50 of the theoretically possible 75 gene-cell line combinations. Each of these biallelic mutations were predicted to cause significant disruption of gene function (Figure 1A, Table S5; Douville et al., 2013; Masica et al., 2017).

### Orthogonal validation of knockout lines

We next sought to orthogonally validate the disruptions in these 302 lines. We established a hierarchical validation strategy where we first sought to establish loss of protein by Western blot analysis, followed by immunohistochemistry (IHC) and finally loss of wild-type transcript by transcriptome analysis. Western blot assays were performed on 95 cell line and protein loss was confirmed in 71 of them (Figure 1B and Table S6). For 102 of the cell lines, we performed IHC assays and confirmed protein loss in 65 lines (Figure 1C and Table S6). Finally, to validate 4 genes lacking western or IHC assays and to begin to characterize the transcriptomes of additional selected lines, we constructed RNA-Seq libraries from 97 isogenic cell lines and sequenced them to an average depth of 2.2 × 10e7 reads per cell line (Table S6). To be validated by RNA-Seq, at least 15 reads (Average = 87.5, N = 19) covering the mutated or flanking exons were required with no evidence of wild-type sequence or in-frame exon skipping. In total, we were able to validate loss of normal gene product at the protein or RNA level in 152 cell lines, representing 20 of the 22 targeted genes (Figure 1D and Table S5). One hundred of these 152 lines were subsequently assembled into the “Cancer Pathway Knockout Panel” to minimize overlap while maximizing diversity (Tables 1 and S7).

As noted above, several genes with known chemical sensitivities were also targeted to provide controls for assay development (Tables S2, S8 and examples in Figure S2). In addition, we exploited the known differential sensitivity of cells without genetic inactivation of *TP53* to the small molecule MDM2 inhibitor Nutlin-3a (Vassilev et al., 2004). Nutlin-3a causes cell senescence or death in cell lines with functional *TP53* by increasing the amount of available p53 protein. As expected, cell lines with wild-type *TP53* were 5–10 times more sensitive to Nutlin-3a than their *TP53* null counterparts (Figure S3).

### Development of a multiplexed ratiometric cell growth assay

**Figure 2:**
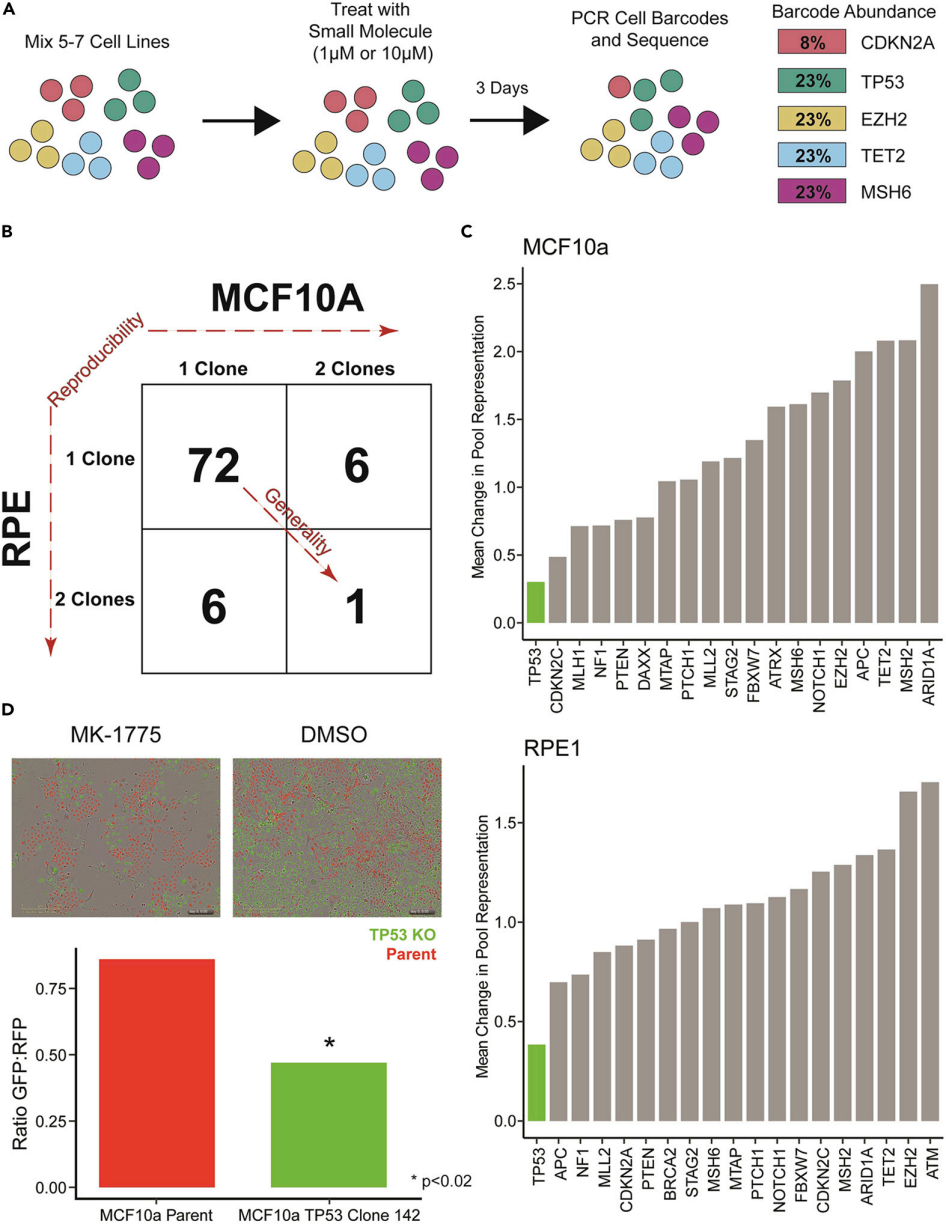
Synthetic lethal screen with FDA-approved small molecules (A) Cartoon example of high-throughput assay design showing hypothetical killing of a *CDKN2A* knockout cell line. (B) Two by two table summary of results for hits from the small molecule screen. (C) Average change in fraction of reads for all knockout cell lines in RPE1 at 10μM and MCF10a at 1μM stratified by knockout gene in the presence of MK-1775. (D) Co-Culture of TP53 wild-type MCF10a parent (RFP) and *TP53* knockout cell line MCF10a TP53 142 (GFP) treated with MK-1775. A quantification of the image is shown as a bar graph.

To demonstrate the potential utility of our engineered TSG knockout panel, we developed a screening platform that permits co-culture of multiple cell lines in a single well. Each of the cell lines in a single well thereby provides multiple internal controls for drugs that are generally toxic, rather than specifically toxic to a cell line harboring a specific disrupted pathway. Primers were designed to universally PCR-amplify every integrated gRNA in our cell line panel. Subsequent sequencing of the amplification products produced unique DNA barcodes for each cell line in a well. The representation of individual barcodes in the sequencing data thereby reflected the number of cells with the particular pathway disruption (Figure 2A). Though we constructed knockouts in three different parental cell lines, we pooled only cell lines derived from one parental cell line in any single well to more easily control for differences in parental cell line growth (Figure 2A).

With this pooling strategy (Figures 2A and S4), we could obtain nearly 15,000 measurements of cell growth from a single lane of an Illumina HiSeq 2500 instrument. This typically represented the output of 22 plates, each containing 96 wells using 5 to 7 cell lines per well. The multiple intra-well and inter-well measurements, along with positive and negative controls in every 96-well plate, provided an interlocking group of ratiometric measurements that enhanced specificity. Indeed, we observed a false positive rate of only 0.11% for the negative control well (DMSO only, no drug).

### High-throughput screen of FDA-approved and clinical trial compounds

Using the multiplex assay described above, we evaluated a library of 2,658 FDA-approved small molecule compounds for their ability to inhibit the growth of cell lines with specific pathway defects (Table S9). For this screen, we used 81 cell lines derived from two different parental cell lines and representing 19 targeted pathways (See Table S5 for cell lines used in screen). Each of the 81 cell line were exposed to 1μM or 10μM of compound for 72 h (Figure 2A). As negative controls, each 96-well plate included wells without drug or vehicle and wells with only vehicle (DMSO). The positive controls included in each 96-well plate were one well treated with Nutlin-3a (an inhibitor of normal p53 function) and one well treated with staurosporine (a non-specific, cytotoxic control). The negative and positive controls performed as expected, with DMSO having no effect on growth (Figure S5A) and staurosprine producing a marked reduction in growth (Figure S5B). The other positive control (Nutlin-3a) documented a pronounced difference between the growth of cell lines dependent on their *TP53* status, as expected (Figure S5C).

In total, 430,596 compound-cell line interactions were scored in this assay. Compounds of interest were identified by requiring both a statistically (i.e., Z-score of −1.5 or less) and biologically significant (i.e., greater than 50% inhibition) effect as described in the STAR Methods section. Furthermore, it was required that this criteria were satisfied by independent cell lines with the same pathway disrupted in two parental cell backgrounds (Figure 2B). After applying these stringent filters to the 2,658 FDA-approved small compounds, 1 hit emerged: *TP53* loss sensitized cells to the effects of MK-1775 (Figures 2B and 2C, Table S10).

To model the power of the screen, we examined the distribution of counts across the entire screen, consisting of 43 RPE1 and 38 MCF10A cell lines and performed an in silico analysis where we reduced the level of each cell line reads by 50% or 90% in a given well (Table S11). This analysis indicated that a 90% reduction in reads would yield a Z-score of −1.5 in 94% of the wells for RPE1 and 79% of the wells for MCF10A. Likewise, a 50% reduction in reads would yield a Z-score of −1.5 in 75% of the wells for RPE1 and 38% of the wells for MCF10A. These results were consistent, and slightly better, than pilot experiments used to determine optimal conditions for this screen (data not shown). Based on these results, we felt the 50% cutoff was reasonable to ensure biological significance while still preserving a reasonable statistical power. Supporting this criteria, the average observed decrease in expression of all wells meeting statistical significance (Z factor < −1.5) was 45% (29%–61% interquartile range).

### TP53 deficiency sensitizes to MK-1775 (AZD1775)

MK-1775 demonstrated selective growth inhibition of *TP53*-deficient cell lines from both RPE1 and MCF10a backgrounds within the primary screen when treated in the low micromolar ranges (Figure 2C). This result was confirmed in an orthogonal fashion using co-cultures of GFP- (*TP53* mutant) and RFP (*TP53* wild type)-labeled isogenic knockout cell lines (Figure 2D). MK-1775 is an inhibitor of *Wee1, a* kinase that controls the G2/M transition (Hirai et al., 2009). Previous studies have indicated that MK-1775 can selectively inhibit the growth of TP53-deficient cells in human cancers *in vivo* and *in vitro* in combination with radiation or chemotherapy and MK-1775 is currently in clinical trials for *TP53*-deficient tumors in combination with chemotherapy or radiation (Center, 2016; Ku et al., 2017; Leijen et al., 2016; Osman et al., 2015; Rajeshkumar et al., 2011). Thus, our result does not represent a new drug discovery but rather represents an unbiased proof of principle for the new assay.

## Discussion

The results present above document two aspects of a novel resource for drug screening. First, we describe a panel of highly characterized isogenic cell lines containing single gene knockouts in critical cancer pathways. Second, we describe a multiplex, sequence-based assay that can be used for drug screening.

One important characteristic of our panel is the extensive validation undertaken for candidate knockout cell lines. Each of them had out-of-frame insertions or deletions which could not be “exon-skipped” without giving rise to a downstream out-of-frame event. Moreover, all cell lines show a lack of functional RNA or protein products. In total, we derived a panel of 100 well-annotated isogenic cell lines that were validated in this way. Not all of the cell lines have to be included in a drug screen, particularly an initial one. But the redundancy inherent in the cell lines described here allows rapid confirmation of the activity of a drug identified in an initial screen. The variety of pathways and cellular backgrounds represented in these lines should provide an ideal resource for phenotypic high-throughput screening for a wide range of disease targets.

### Limitations of the study

This study was performed in non-cancerous cell lines from tissues of origin that may not best reflect the biology of the targeted cancer pathways. Not all targeted genes were represented by two or more knockouts in every background which may make results for these genes less reproducible than originally desired.

## STAR★Methods

### Key resources table

**Table:** 

REAGENT or RESOURCE	SOURCE	IDENTIFIER
**Antibodies**		

Antibodies for confirming knockout status	Various	Table S12

**Chemicals, peptides, and recombinant proteins**		

High throughput screening library	Selleck Chem	Table S9

**Deposited data**		

RNA-seq data fastq files	EGA	EGAD00001008559
Primary results from the high throughput screening	Mendeley	Wyhs, Nicolas (2022)

**Experimental models: Cell lines**		

RPE1	ATCC	CRL-4000
MCF10A	ATCC	CRL-10317
RPTec	ATCC	CRL-4031
HEK293	ATCC	CRL-1573
Knockout cell lines	This Paper	Table S5

**Oligonucleotides**		

CRSPR gRNA sequences	This paper	Table S3
CRPSR target sequencing primers	This paper	Table S4

**Software and algorithms**		

SQL	Microsoft	https://www.microsoft.com/en-us/sql-server/sql-server-2019
HISTAT2	Pertea et al. (2016)	http://daehwankimlab.github.io/hisat2/
StringTie	Pertea et al., 2015	https://ccb.jhu.edu/software/stringtie/
Ballgown	Pertea et al. (2016)	https://www.bioconductor.org/packages/release/bioc/html/ballgown.html
R	R Core Team (2021)	https://www.R-project.org/

### Resource availability

#### Lead contact

Further information and requests for resources and reagents should be directed to and will be fulfilled by the lead contact, Kenneth Kinzler Ph.D (kinzlek@jhmi.edu).

#### Materials availability

Cell lines generated in this experiments can be obtained by contacting the Johns Hopkins Genetic Resources Core Facility (GRCF) Biorepository@jhmi.edu.

### Experimental model and subject Details

#### Cell lines & cell culturing

RPE1, HEK293, MCF10a, and RPTec cells were purchased from The American Type Culture Collection (Virginia, USA). RPE1 cells (ATCC CRL-4000) are female in origin and were grown in RPMI 1640 Medium (Invitrogen, California, USA, Cat #11875-119) supplemented with 10% fetal bovine serum (HyClone, Utah, USA, Cat #16777-006). RPTec cells (ATCC CRL-4031) are male in origin and were grown in EPITHELIAL CELL MEDIUM-Complete Kit (Science Cell Research, California, USA, Cat #4101). HEK293 (ATCC CRL-1573) was grown in DMEM (Thermo Fisher, USA, Cat# 11995065) supplemented with 10% FBS (HyClone, Utah, USA, Cat #16777-006), MCF10a cells (ATCC CRL-10317) are female in origin and were grown in Bullet Kit MEBM Basal Medium 500 mL with cholera toxin (Sigma C8052-2MG) at 100 ng/mL and MEGM SingleQuots Kit Suppl. & Growth Factors (Thermo Fisher, USA, Cat# CC3150) excluding gentomycin. *In vitro* all cells were grown at 37°C with 5% CO_2_. Mycoplasma testing performed by The Genetic Resources Core Facility at Johns Hopkins University School of Medicine (Maryland, USA). Cells were not authenticated internally as they were sourced from a non-profit company (ATCC).

The cell line characterized in this study are detailed in Table S5. A subset of the lines were validated and banked for distribution including 100 lines targeting the 19 critical cancer pathways (Table S7) and 8 lines where control non-cancer pathway were targeted (Table S8). Of the non-tumor suppressor genes targeted, *MTAP* is of particular interest and represented by multiple lines because it is frequently co-deleted with *CDKN2A* making it passenger mutation targetable for therapeutic benefit in human cancers (Mavrakis et al., 2016).

### Method Details

#### CRISPR-Cas9

Integrated CRISPR-Cas9 gRNAs were designed using Chop-Chop based upon common mutations sites identified in COSMIC (Montague et al., 2014). Each gene was targeted with 6–12 gRNAs (Table S1). gRNAs were ordered from IDT Technologies (Iowa, USA) with the addition of ligation sequences: caccgNNNNNNNNNNNNNNNNNNNN and aaacNNNNNNNNNNNNNNNNNNNNc. gRNAs were ligated into the LeniCRISPR V2 plasmid (Addgene, Massachusetts, USA, Cat #52961) using previously published protocol (Sanjana et al., 2014). CRISPR/Cas9 plasmid was virally transduced into cells using Lenti-X Packaging Single Shots (VSV-G) using manufacturer’s instructions (Clontech, California, USA, Cat #631275). See Table S3 for list of all gRNAs utilized. Any target genes which appear in the gRNA list but do not have a corresponding cell line clone associated with it were unable to be obtained. This was due to a variety of reasons, including but not limited to loss of the gene being lethal.

#### Mutation detection and analysis

DNA was extracted from cells using Quick Extract (Lucigen, Wisconsin, USA, Cat #QE09050) and amplified using primer pairs listed in Table S4 designed to amplify 66–80 base pair segments containing the predicted cut site for each of our gRNAs listed in Table of gRNAs. Primer sets were designed for the SafeSeqS application, and were sequenced on an Illumina MiSeq and analyzed as previously described (Cohen et al., 2018).

#### Western Ab information

Cells were lysed using RIPA buffer (Thermo Fisher, USA, Cat #89901) with x1 protease inhibitor (Thermo Fisher, USA, Cat #4693159001) and left on ice for 30 min. Samples were then centrifuged at max speed for 3 min in a QIAshredder (Qiagen, Maryland, USA, Cat #79654) before being transferred to a new Eppendorf tube. Protein was quantified using a BCA assay (Thermo Fisher, USA, Cat #23227).

Westerns were performed by loading 30–50ug of total protein per well into 15 well polyacrylamide gels (Bio-Rad, California, USA, Cat #456-1086) and run for 30 min at 200 V. Gels were then transferred using manufacturer’s instructions (based on size) to nitrocellulose membrane using a Bio-Rad turbo transfer apparatus. Membranes were blocked for 1 h with 3% milk TBS-Tween before being incubated overnight in primary antibody (concentration dependent on antibody). Membranes were then washed 4 times for 5 min each with TBS-Tween. Secondary antibody was applied at 1:2500 (Abcam, United Kingdom, anti-mouse Cat #ab6728 or anti-rabbit Cat #ab6721). Membranes were imaged using Pierce™ ECL Western Blotting Substrate (Thermo Fisher, USA, Cat #32106) following manufacturer’s instructions and imaged on a Bio-Rad Chemidoc (Bio-Rad, California, USA). Table S12 indicated antibodies used for westerns and their typically employed concentrations.

#### Immunohistochemistry (IHC)

Antibody concentrations for IHC were specific for each protein being screened (Table S9). Generally, Immunolabeling for a protein was performed on formalin-fixed, paraffin embedded sections on a Ventana Discovery Ultra autostainer (Roche Diagnostics, Switzerland). Briefly, following dewaxing and rehydration on board, epitope retrieval was performed using Ventana Ultra CC1 buffer (Roche Diagnostics, Switzerland, Cat #6414575001) at 96°C for 64 min. Primary antibody was applied at 36°C for 60 min. Primary antibodies were detected using an anti-rabbit HQ detection system (Roche Diagnostics, Switzerland, Cat #7017936001, #7017812001) followed by Chromomap DAB IHC detection kit (Roche Diagnostics, Switzerland, Cat #5266645001), counterstaining with Mayer’s hematoxylin, dehydration and mounting.

#### RNA seq methods

For RNA extraction, cells were pelleted, frozen in liquid nitrogen, and stored at −80°C until RNA extraction. RNA extraction using Qiagen AllPrep DNA/RNA Mini Kit (Qiagen, Maryland, USA, Cat# 80204) per manufacturer’s instruction with cell homogenization in RLT buffer via QIAshredder (Qiagen, Maryland, USA, Cat# 79656). RNA quality control using Agilent Tapestation 2200 (Agilent, California, USA, Cat# G2964AA) and the Agilent RNA ScreenTape (Agilent, California, USA, Cat# 5067- 5576) and Agilent RNA ScreenTape Sample Buffer and Ladder (Agilent, California, USA, Cat# 5067- 5577, Cat# 5067- 5578) per manufacturer’s instruction. Library prep using Illumina RNA library prep kit (Illumina, California, USA, Cat # RS-122-2001) and sequenced on an Illumina HiSeq 4000 paired end using manufacturer’s instructions.

Sequencing reads aligned to Hg38 using HISAT2 (version 2.0.5), assembly and quantification was performed using StringTie (version 1.3.3) and differential expression was performed using R package Ballgown (version 2.6.0) as described (Pertea et al., 2015, 2016). Exon skipping was determined using IGV Viewer Sashimi Plots (Robinson et al., 2011).

#### Hit validation – Fluorescent labeled cell lines & cell confluence

*TP53* knockout cell lines were labeled with either a GFP or RFP plasmid (Essen Bioscience, USA, Cat #4477 and #4478). We then performed a co-culture of each knockout cell line with its respective parental line and treated with the compound of interest in a dose response curve. We imaged cells every 6 h for 4–6 days of treatment using Incucyte Zoom (Sartorius, Michigan, USA). Fluorescence was quantified using four locations in each treated sample by the IncuCyte Zoom 2016B software. Confluence assays were plated in 96-well plate format and imaged every 6 h for 4–6 days of treatment using Incucyte Zoom.

#### Hit validation – Sybr Green cell counting assay

Cell response assays were quantified using a sybr readout. Cells were rinsed 2× in phosphate buffered saline (PBS) (Thermo Fisher, USA, Cat#10010-049) and lysed with 50 μL of 0.2% SDS (Thermo Fisher, USA, Cat#15553027) for 2 h at 37°C. 150 μL of Sybr Green I (Thermo Fisher, USA, Cat#S7563) solution (1:750 in water) was added and mixed 10× with a pipette. Fluorescence was read out with 485 nm excitation and emission measured at 530 nm on a BioTek microplate reader (BioTek, Vermont, USA). DNA content of each sample analyzed relative to untreated samples.

### Quantification and statistical analysis

#### Statistical testing

Z-scores calculated utilizing the ratio of target cell UID to total sequencing reads in a well

$$z=\frac{\left(\text{well}\;\text{abundance}\;\text{ratio}\right)-\left(\text{median}\;\text{plate}\;\text{abundance }\;\text{ratio}\right)}{\left(\text{stdev}\;\text{plate}\;\text{abundance}\;\text{ratio}\right)}$$

#### Small molecule cell line screen

We identified optimal plating of cells up to 7 distinct cell lines from the same background and 5,000 total cells per well of a 96-well plate maintained the best cell line representation and compound response. The library chosen was the FDA-approved & Passed Phase I Drug Library in 96 well format (Selleck Chem LLC, Houston TX, #L3800). We screened 81 total knockout cell lines across MCF10a and RPE1 cell line backgrounds and 19 critical cancer pathways at two doses: 1μM and 10 μM. Cells were plated in the morning and treated with the compound libraries in the evening of day 1. Plates were harvested on day 4 and molecular barcodes identifying each cell line were quantified by high-throughput sequencing.

Sequencing using barcoded forward primer including Illumina primer sequence, N14, plate barcode, and LentiV2 sequence (AATGATACGGCGACCACCGAGATCTACACTCTTTCCCTACACGACGCTCTTCCGATCT NNNNNNNNNNNNNN**BARCODES**TGTGGAAAGGACGAAACACC). Reverse primer includes Illumina primer sequence, well barcode, spacer, and a LentiV2 sequence (CAAGCAGAAGACGGCATACGAGAT**BARCODES**NNCGGACTAGCCTTATTTTAACTTGC). This amplicon requires 96 reverse primers and 1 forward to amplify and uniquely identify each well in a 96-well plate (Figure S4). In total 25 plate and 192 well barcodes were designed and verified. These primers were used to amplify cell pools after DNA extraction using Quick Extract (Lucigen, Wisconsin, USA, Cat #QE09050). Amplified reads were sequenced on either an Illumina MiSeq or Illumina HiSeq 2500.

Screen controls were scored by evaluating the ratio of unique identifier (UID) reads matching a single cell line in each staurosporin treated well to the cell lines respective UID reads from the untreated DMSO wells in the same screen plate. This was performed for each knockout line within each screen plate. To assess cell line representation and performance in each treated well, we calculated the z-score for each cell line’s fraction of reads within a single well to compare cell line abundance in drug treated wells and compared to the 95 other wells within the same plate. We assume the null hypothesis – for any given compound, it will not have a specific interaction with our gene of interest. Compounds in our screen with UIDs less than two-fold more unique molecular barcodes than the non-specific small molecule control within the same plate, staurosporine, were classified as non-specific cell killing. We did not consider compounds from these wells in our analysis.

To determine the z-score threshold we looked at representation of each cell line in the DMSO treated control wells and down-sampled the sequencing of these well to 0 in increments of 10%, calculating the z-score at each increment to determine the power to see each cell line. This was determined for each plate in our screen and thresholds determined from the 3^rd^ quartile, the lowest 25% of plates based on cell line representation within the plate. Based on this in silico calculated 3^rd^ quartile z-score we classified cell lines as well powered, and used a cutoff of −1.5, or low powered, and used a cutoff of −1.0 to identify compounds of interest. Based on this classification, a maximum z-score threshold was set for each cell line either −1.5 for well powered or −1 for low powered. All compound-cell line z-scores below these thresholds were considered compounds of interest. For a compound to be considered a hit, the majority of cell lines in two cell line backgrounds would need to identify it as a compound of interest. Applying these criteria to DMSO wells showed that 0.11% of controls wells met the hit criteria.

## Data Availability

• RNA-seq data fastq files have been deposited at EGA under accession number EGAD00001008559 and are publicly available as of the date of publication. • Primary results from the high throughput screening is available on Mendeley (Wyhs, Nicolas (2022), “Cook et al. Primary Isogenic Cell Line Screen Data 2022”, Mendeley Data, V1, https://doi.org/10.17632/rz47vn4v2d.1). • Any additional information required to reanalyze the data reported in this paper is available from the lead contact upon request.

## Supplemental information

Document S1. Figures S1–S5

Table S1. Fraction of tumors with somatic mutations in targeted pathway, related to Figure 1

Table S2. Control genes and cancer pathway with known small molecule sensitivities, related to Figure 1

Table S3. Genes and gRNA used to target pathways, related to Figure 1

Table S4. Sequencing primers used to identify CRISPR targeting, related to STAR Methods

Table S5. List of cell lines with successful CRISPR targeting∗, related to Figure 1

Table S6. Gene KO confirmation method, related to Figure 1

Table S7. Cell line panel of validated knockouts targeting 19 critical cancer pathways∗, related to Figure 1

Table S8. Cell line panel of validated knockouts targeting noncancer control genes∗, related to Figure 1

Table S9. Selleck chem library, related to Figure 2

Table S10. HTS results compounds of interest, related to Figure 2

Table S11. Downsampling of screen data, related to STAR Methods

Table S12. Antibodies used for validation, related to STAR Methods
