# Supplementary material for: An isogenic cell line panel for sequence-based screening of targeted anticancer drugs
Source: iScience. 2022 May 23;25(6):104437. doi: 10.1016/j.isci.2022.104437 (PMC9184558; doi:10.1016/j.isci.2022.104437)
Supplement: Document S1. Figures S1–S5 [file mmc1.pdf]

iScience, Volume *25*

Supplemental information

An isogenic cell line panel for sequence-based screening of targeted anticancer drugs

Ashley L. Cook, Nicolas Wyhs, Surojit Sur, Blair Ptak, Maria Popoli, Laura Dobbyn, Tasos Papadopoulos, Chetan Bettegowda, Nickolas Papadopoulos, Bert Vogelstein, Shibin Zhou, and Kenneth W. Kinzler

Supplemental Figures and Data

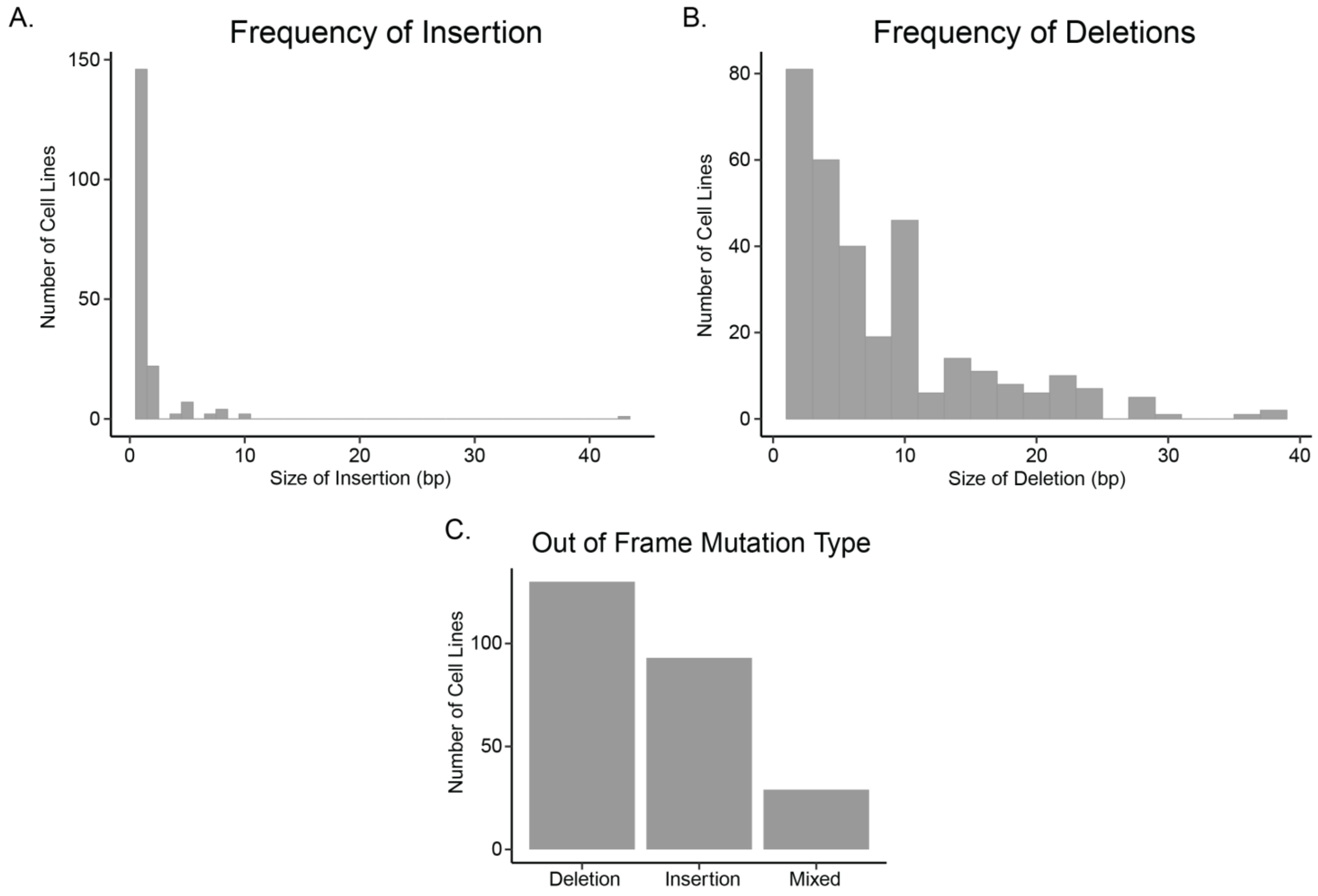

Supplemental Figure S1: Histogram of insertions related to figure 1 A. and deletions B. present in cell lines with biallelic out of frame mutations identified by SafeSeqS. The x-axis shows the number of bases inserted or deleted in bins of 2bp. C. Number of cell lines containing specific mutation types. Mixed denotes cell lines with an out of frame deletion in one allele, and out of frame insertion in the other.

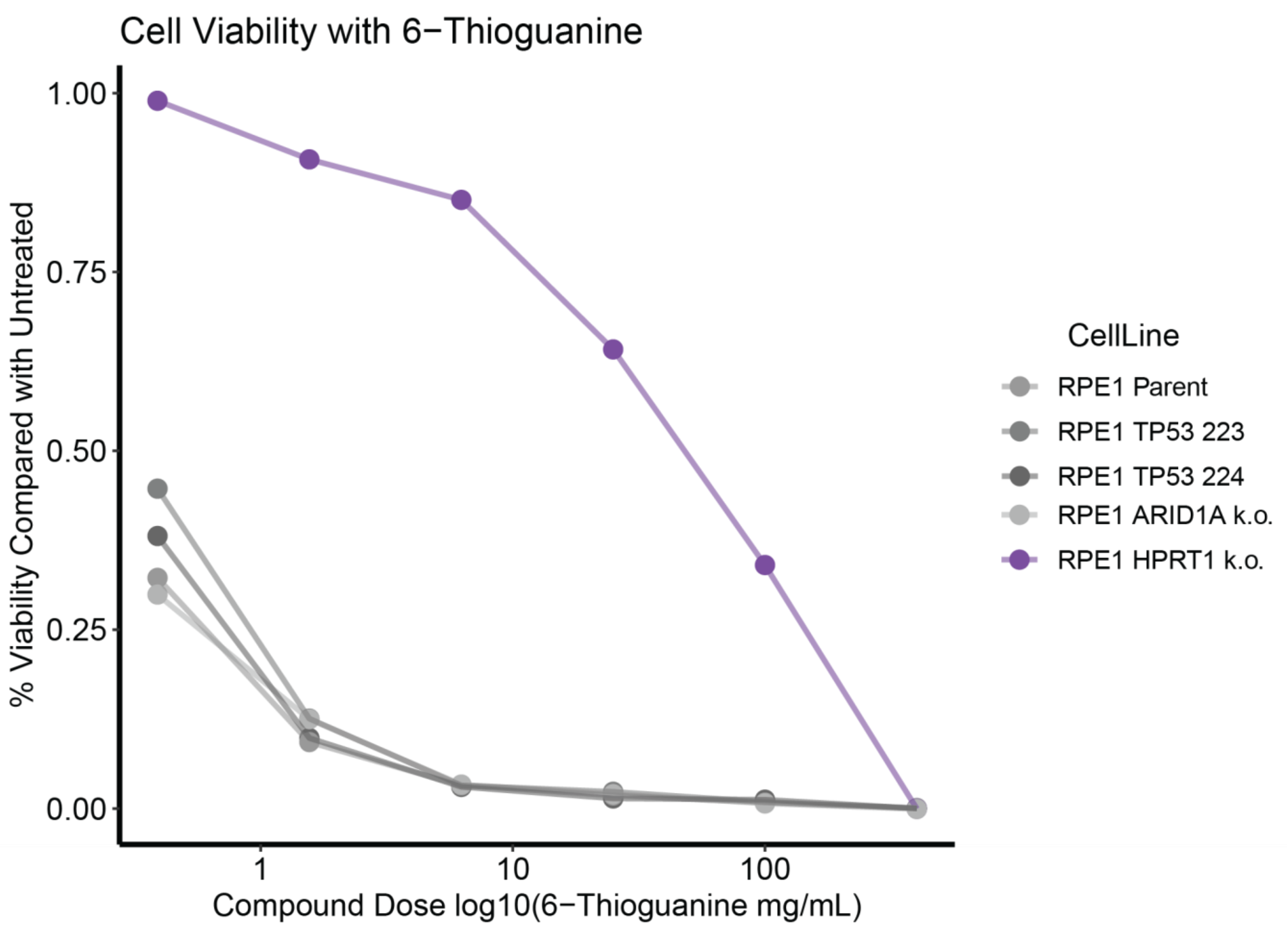

Supplemental Figure S2: *HPRT1* and *TP53* Knockouts from the RPE1 background treated with 6-Thioguanine (6-TG) for 3 days and readout by SYBR green assay (DNA content). Related to figure 1.

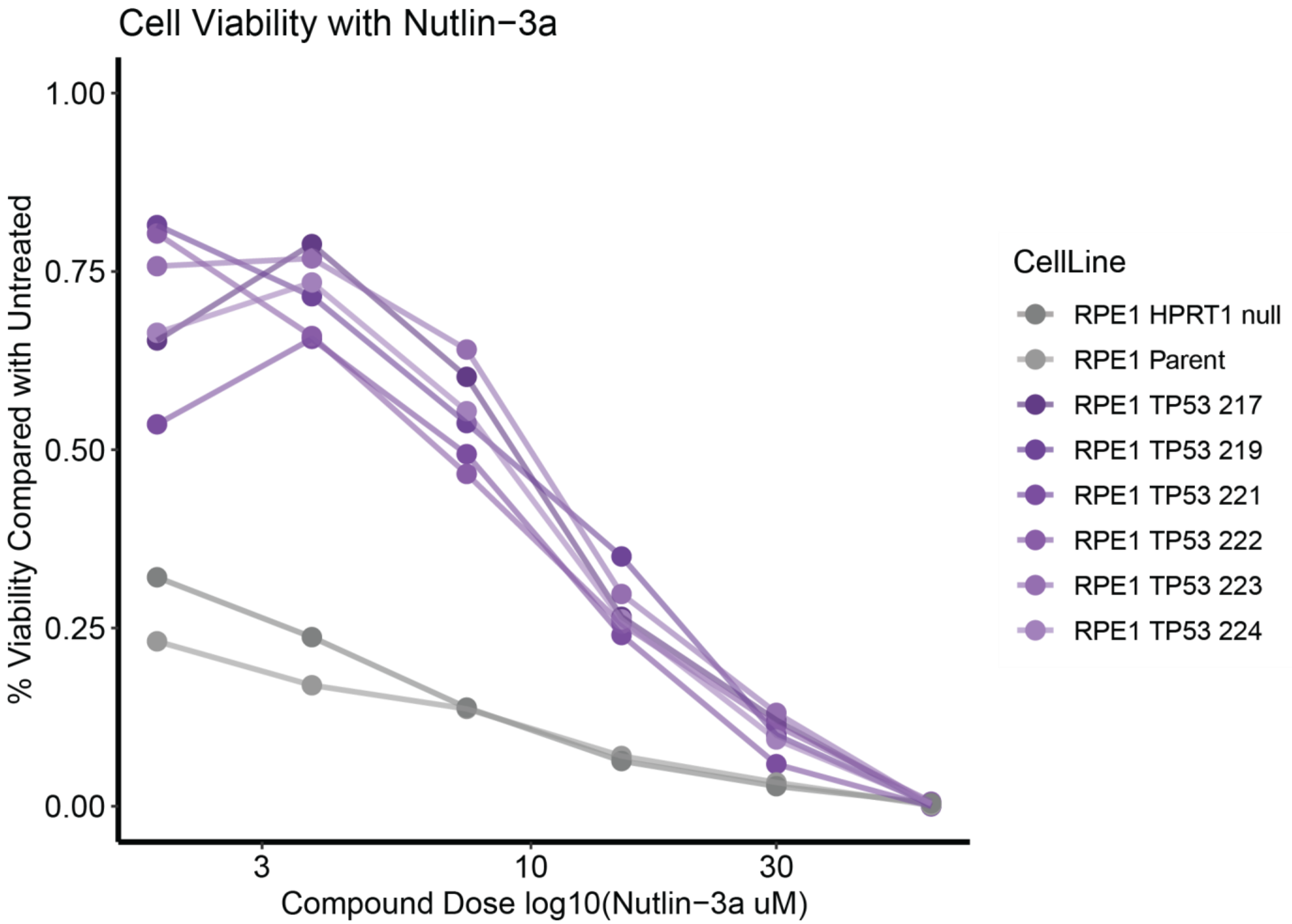

Supplemental Figure S3: Dose response of RPE1 *TP53* knockout cell lines compared with RPE1 parent and RPE1 *HPRT1* knockout cell lines to Nutlin-3a, treated over 3 days and readout by SYBR green assay (DNA content). Related to figure 1.

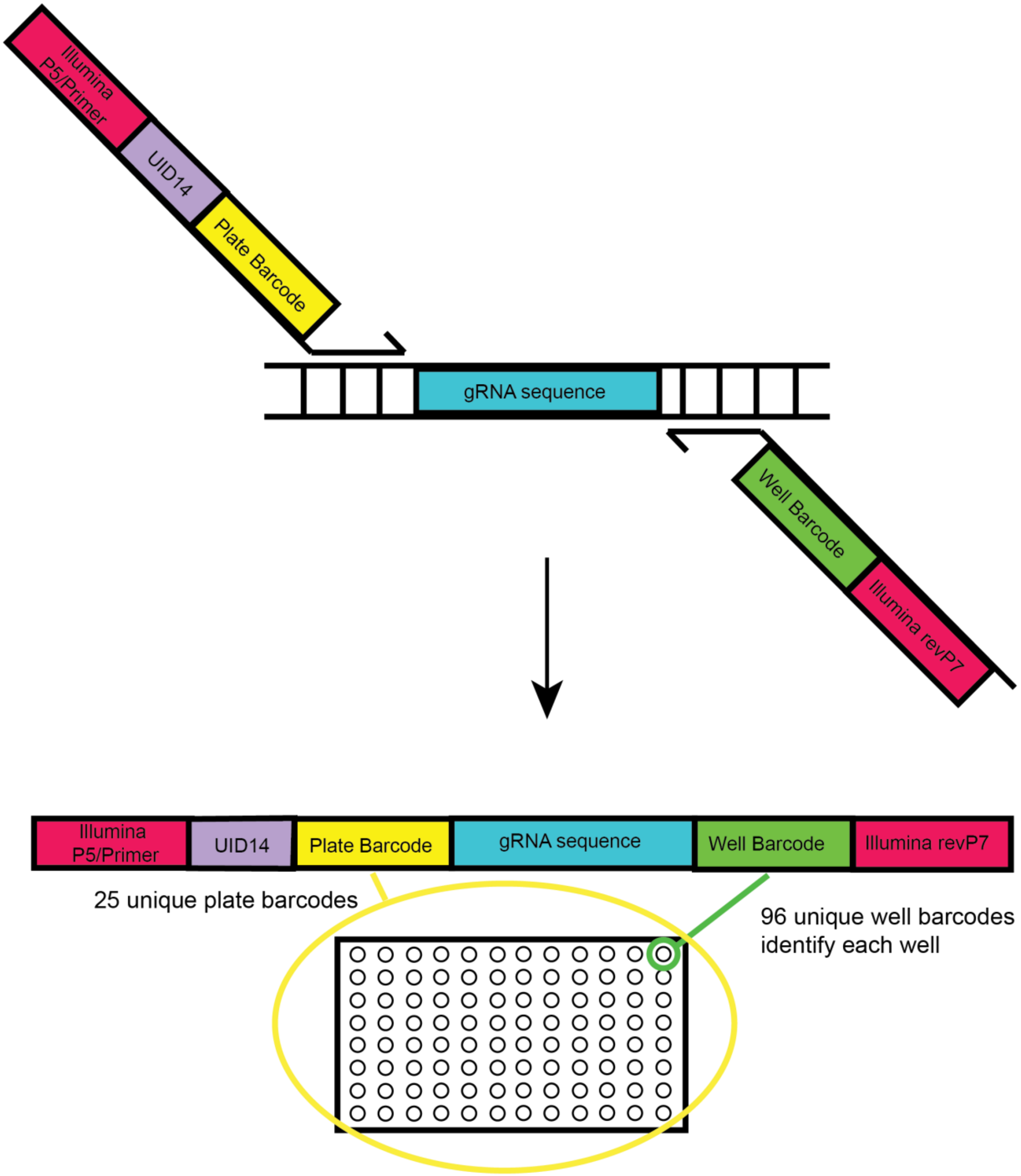

Supplemental Figure S4: Cartoon showing amplification strategy of integrated barcode (gRNA) for high throughput sequencing, related to figure 2. Up to 7 clones were per well were co-amplified with the above strategy at a time (each clone contains a unique barcode gRNA). The amplicon is 197bp in size.

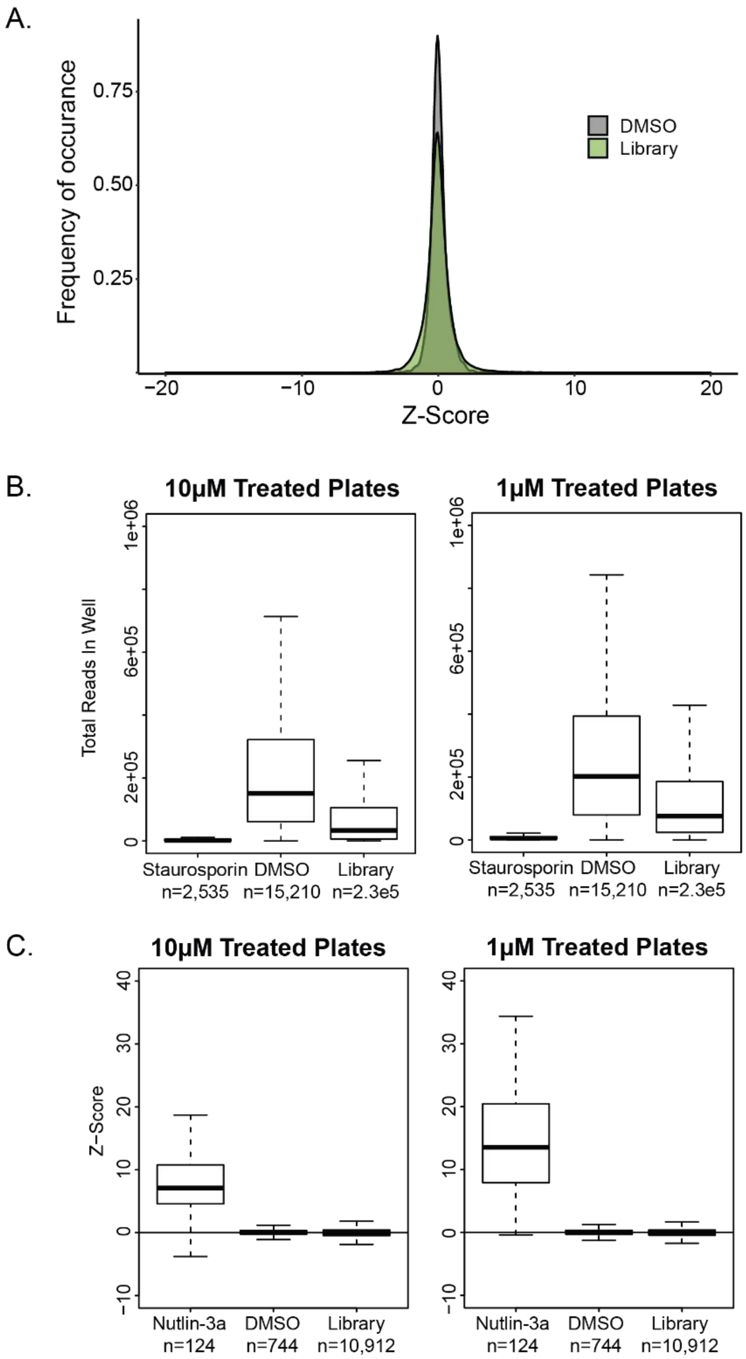

Supplemental Figure S5: Screening summary, related to figure 2. A. Distribution of Z-Factors in the Selleck Chem HTS for DMSO control wells and wells treated with a compound (library). B. Boxplot showing total number of unique identifier reads in control or library containing wells. C. Boxplot showing distribution of Z-Factors for *TP53* knockout clones when treated with DMSO, library or nutlin-3a. Z-Factors were calculated based on plate based median and standard deviation, excluding control wells.
